# Effect of pretreatment with a small dose of esketamine on sufentanil-induced cough during anesthesia induction: a randomized controlled trial

**DOI:** 10.1186/s12871-024-02501-0

**Published:** 2024-03-26

**Authors:** Liangliang Gao, Zhuoliang Zhang, Yi Zhu, Xinyu Lu, Yue Tian, Lei Wei

**Affiliations:** 1 Jiangsu Province Key Laboratory of Anesthesiology, Xuzhou Medical University, Xuzhou, 221000 China; 2 Suzhou Hospital Affiliated to Nanjing Medical University, Suzhou, 215000 China

**Keywords:** Esketamine, Sufentanil, Cough, Anesthesia

## Abstract

**Background:**

Sufentanil-induced cough is common during the induction of anesthesia. The objective of this study was to determine whether pretreatment with a small dose of esketamine is effective in treating sufentanil-induced cough.

**Methods:**

220 patients were screened, and 200 patients who had scheduled elective surgery and were between 18 and 70 years old were randomly divided into two groups. Before sufentanil was administered, esketamine group (group K) was injected with 0.15 mg/kg esketamine at 5 s, and control group (group C) was administered with the same volume. Within 1 min after sufentanil(0.4ug/kg) injection during induction, cough incidence and severity were evaluated. After sufentanil was injected, we recorded its hemodynamic changes and side effects.

**Results:**

In the esketamine group (group K) and control group (group C), there was an incidence of cough of 5 and 34%, respectively. The esketamine group (group K) had a significantly lower incidence and severity of cough compared to the control group (group C) immediately after sufentanil injection (*P* < 0.05). MAP and HR did not differ significantly between the two groups during three different times of general anesthesia induction (*P* > 0.05).

**Conclusion:**

In our study, we found that sufentanil-induced cough was significantly reduced by pretreatment with 0.15 mg/kg esketamine, but with no significant changes in the hemodynamic status.

**Trial registration:**

Chinese Clinical Trial Registry (ChiCTR2200063821, registered date: 17/09/2022), http://www.chictr.org.cn

## Background

The frequent use of sufentanil as an induction agent is due to its strong analgesic properties, short duration of action, and cardiovascular stability. However, The cough caused by sufentanil can occur during anesthesia induction. The prevalence of sufentanil-induced cough (SIC) has been recorded by various studies at 15 to 45% [1, 2]. Although SIC is temporary in most cases, it can cause an increase in intracranial, intraocular, and intra-abdominal pressure, which lead to disastrous consequences in patients with a compromised central nervous system(CNS), open eye injury and cardiovascular diseases [3, 4]. A variety of studies have been carried out to find ways to prevent cough caused by sufentanil during anesthesia induction.

Various approaches have been proposed to suppress SIC, such as pretreatment with dexmedetomidine [4], dezocine [5], nalbuphine [6], or lidocaine [7], as well as with non-pharmacological approaches like central venous injection of sufentanil [1] and extend injection time [8]. However,the interventions should not be extensively implemented in clinical practice due to various aspect results such as long onset time, bradycardia, and respiratory depression. Thus, figuring out proper drugs to suppress SIC is urgently needed. Strong analgesic and bronchodilatory effects are known to be associated with the NMDA antagonist ketamine, which has also been demonstrated in a previous study to lower the incidence of fentanyl-induced cough [9]. Esketamine, which is an inhibitor of the NMDA receptor, is commonly used to treat pain and depression, and has more powerful pain-relieving effects and fewer adverse events than ketamine [10]. To our knowledge, there is no study that evaluates the efficacy of esketamine for cough caused by sufentanil. Therefore, the aim of this study was to examine the effectiveness of esketamine in treating cough caused by sufentanil.

## Methods

### Study design and setting

This single center randomized controlled clinical trial was conducted at the First Affiliated Hospital of Soochow University between March 2023 and August 2023. This study was approved by the Istitutional Research Ethics Committee of the First Affiliated Hospital of Soochow University, Suzhou, Jiangsu, China. The trial was registered in the Chinese Clinical Trial Registry (No. Chi CTR2200063821). Each patient was given written informed consent in accordance with the principles of the Helsinki Declaration. CONSORT guidelines are adhered to by this study.

### Participants

A total of 220 patients were screened during the study conducted at the First Affiliated Hospital of Soochow University. This study included 200 participants who were scheduled for elective surgery. ASA I-II patients from both sexes and aged 18–70 years were included. The exclusion criteria included BMI ≥ 30 kg/m^2^, patients with severe liver and kidney dysfunction, history of chronic cough, bronchial asthma, chronic obstructive pulmonary disease, acute upper respiratory tract infection, recently used bronchodilators, steroid hormones, or angiotensin converting enzyme inhibitors, and patients who were allergic to the study drug, or had a history of mental illness.

### Randomization and blinding

Eligible participants were randomized 1:1 to group K, which received esketamine, and group C, which received normal saline, using computer-generated random numbers. In sealed opaque envelopes, the randomization results were stored prior to medication preparation. Opaque envelopes that were sealed held patient group information. The patients and anesthesiologists who measured the severity of cough were blinded for the group assignment. In a 20-ml syringe, the pretreatment drugs were prepared by an anesthetic nurse who was not involved in inducing anesthesia. Intention-to-treat analysis was applied in this investigation. The data was analyzed based on the original groups to which they were assigned at the end of the study.

### Protocol

Routine monitoring, which included non-invasive blood pressure (NBP), electrocardiogram (ECG), and pulse oxygen saturation (SPO2) was completed upon arrival in the operating room without premedication being administered. The patients were given oxygen and the study drug before anesthesia was administered. Before sufentanil induction, group K pretreated with 0.15 mg/kg esketamine for 5 s, while group C pretreated with the same volume of normal saline. After pretreatment drug administration, sufentanil was used to induce anesthesia over 5 s, and patients were monitored for episodes of cough 1 min after the injection of sufentanil. The severity of the cough was classified as mild (1–2 times), moderate (3–5 times), and severe (> 5 times) depending on the number of coughs observed [11]. The primary outcomes were the incidence and severity of the cough 1 min after sufentanil injection. The secondary outcomes included hemodynamic changes at different time points and side effects of sufentanil during general anesthesia induction. Before esketamine or normal saline administration (T0), MAP and HR need to be recorded, followed by 1 min after the administration (T1) and one minute after sufentanil injection (T2). Propofol 2 mg/kg and rocuronium 0.8 mg/kg were used to finish anesthesia induction, followed by orotracheal intubation. 1–2 Mac of sevoflurane with 40% oxygen and 60% air was used to maintain anesthesia for all patients and monitored for at least 30 min after surgery in the PACU. Side effects of sufentanil including apnea, muscle rigidity, bradycardia, or nausea were also recorded during the investigation.

### Sample size determination

The PASS 11 program (PASS, Kaysville, UT, USA) was used to estimate the sample size. With pretreatment with esketamine, the incidence of cough evoked by 0.4 μg/kg sufentanil in our pilot trial was reduced to 8%. The sample size was determined to be 91 patients per group at a power of 80% and a two-tailed α error of 5%. We recruited 100 patients for each group in order to account for potential 10% dropout rates.

### Statistical analysis

Statistical analysis was conducted using SPSS 22.0 software (IBM Corp, Armonk, NY, USA). Normal distribution of the data was confirmed using Kolmogorov–Smirnov test. The mean ± standard deviation is used to express quantitative data with a normal distribution, and an independent-samples T test was used to compare the differences between two groups. The chi-square test was used to analyze categorical variables that were expressed as percentages. Analysis of variance with repeated measures design was used to compare continuous variables at various time periods. It was considered statistically significant when *P* < 0.05.

## Results

**Fig. 1:**
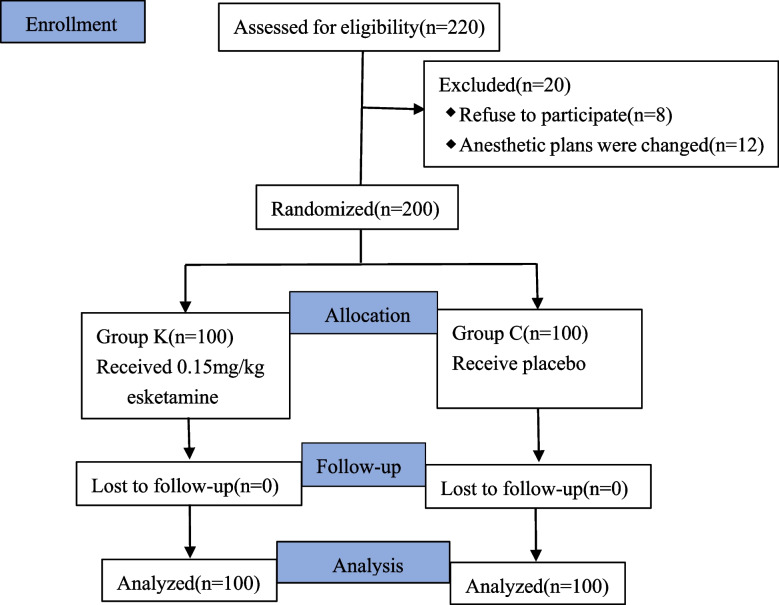
Flow chart of patients participating in this study

After screening 220 suitable patients in total, 200 of them took part in the trial and made it into the final analyses (eight patients rejected to participate, and twelve patients' anesthetic plans altered before the study began) (Fig. 1).

The demographic features of the two groups did not differ significantly (*P* > 0.05), as shown in Table 1.

**Table 1:** Demographics of the two study groups

	Group C	Group K	*P*
Sex (male/female)	52/48	41/59	0.119
Age (years)	42.26 ± 8.24	43.94 ± 10.90	0.506
Height (cm)	165 ± 7.35	166 ± 7.23	0.637
Weight (kg)	62.27 ± 8.58	60.76 ± 7.02	0.858
ASA (I/II)	38/62	43/57	0.471
Type of surgery (n%)			0.755
Gynecological surgery	24(24%)	27(27%)	
Thyroid surgery	16(16%)	21(21%)	
Gastrointestinal surgery	19(19%)	17(17%)	
Thoracic surgery	15(15%)	12(12%)	
Urological surgery	12(12%)	10(10%)	
Orthopedic surgery	14(14%)	13(13%)	

Values are expressed as the mean ± standard deviation or as n patients (%). No significant differences were observed between groups in sex, age, height,weight, ASA class and type of sugery

*C* Control, *K* Esketamine, *ASA* American Society of Anesthesiologists physical status

Group C had a cough incidence of 34%, while group K had a cough rate of 5%. Within a minute following sufentanil injection, the K group experienced a significantly lower incidence and severity of coughing (*P* < 0.05) compared to the C group (Table 2).

**Table 2:** Incidence and severity of sufentanil induced cough

	Group C	Group K	*P*
Incidence (n%)	34(34%)	5(5%)	0.000
Severity (n%)			
Mild	10(10%)	2(2%)	0.017
Moderate	16(16%)	3(3%)	0.002
Severe	8(8%)	0	0.004

Values are expressed as numbers (percentages). Both the incidence and severity of cough in patients in Group K were significantly lower than those in patients in Group C (*P* < 0.05). *K* Esketamine, *C* Control. The severity of cough was graded as mild (1, 2), moderate (3, 5), or severe (> 5) based on the number of cough observed 1 min after sufentanil injection

Between the two groups, there was no significant change in the HR or MAP at any of the three time points (*P* > 0.05, Tables 3 and 4).

**Table 3:** Changes in MAP at different time points

Groups	MAP(mmHg)		
	T0	T1	T2
Group C	95.78 ± 11.20	93.39 ± 13.19	90.28 ± 10.83
Group K	96.91 ± 10.17	95.48 ± 11.45	92.70 ± 12.76
*P*	0.853

**Table 4:** Changes in HR at different time points

Groups	HR(bpm)		
	T0	T1	T2
Group C	73.56 ± 14.24	74.11 ± 15.19	69.13 ± 13.53
Group K	74.91 ± 12.13	76.64 ± 11.25	72.61 ± 10.76
*P*	0.384		

Values are expressed as the mean ± standard deviation. There were no significant differences in either MAP or HR between the two groups. *K* Esketamine, *C* Control, *T0* Time before administration of esketamine or normal saline, *T1* 1 min after administration, *T2* 1 min after sufentanil injection, *MAP* Mean arterial pressure, *HR* Heart rate

Two patients in the control group experienced bradycardia, and one patient experienced nausea. Nevertheless, the esketamine group did not have any negative effects during the trial.

## Discussion

In comparison to the control group, the study's findings showed that pretreatment with 0.15 mg/kg esketamine considerably reduced the occurrence and intensity of cough.

Currently, intravenous injections of sufentanil, fentanyl, and other opioid analgesics during the induction phase of general anesthesia exhibit potent analgesic effects with negligible hemodynamic consequences [12, 13]. However, coughing during the induction of general anesthesia is one of the numerous common side effects of opioids [14]. Sufentanil-induced cough was observed in 34% of the participants in this investigation. Shi et al. [2] found that the incidence of cough was 41.7% after the injection of sufentanil 0.4 μg/kg within 5 s, while in another study by He et al. the incidence of cough was 32% after the injection of sufentanil 0.35 μg/kg within 2 s [1]. A. Agarwal et al. [8] demonstrated that after five seconds of receiving a 0.3 μg/kg injection of sufentanil, the incidence of sufentanil-induced cough could reach 15.8%.The route of administration, medicine concentration, administration velocity, and patient circumstances may all contribute to the varying occurrence rates across different trials [15].

However, the mechanism of sufentanil-induced cough is unclear. Numerous explanations have been put up to explain opioid-induced coughing. A plausible explanation could be that fentanyl triggered the production of histamine in the bronchoalveolar tissue, histamine boosted the excitability of the rapidly adapting receptors through H1 receptors, and the rapidly adapting receptors intensified the cough reflex [16]. Moreover, fentanyl analogs may cause coughing and bronchoconstriction by blocking central sympathetic outflow and stimulating vagus nerve activity [17]. Additionally, it is believed that opioid-induced cough is caused by a pulmonary chemoreflex that is mediated by pulmonary C-fibers or by irritant receptors [18]. Opioids can also bind to and activate μ1 receptors, which is how they function as ε receptor agonists and analgesics. But opioids can also attach to the μ2 receptor, which can result in unpleasant effects like coughing, nausea, and vomiting, as well as respiratory depression [19].

Citric acid is another factor that contributes to opioid-induced cough in addition to opioids themselves. Animals frequently have their cough reflex triggered by citric acid, which stimulates C-fibers in the airway [20]. Opoids are mostly prepared in the form of citrate. The difference in the incidence and severity of coughs among various opioids may be related to the content of citrate in opioids.

It has been demonstrated that a number of methods, including dexmedetomidine, dezocine, lidocaine, and swallow action, can effectively suppress opioid-induced cough. Sun et al. [24] revealed that pretreatment with 0.5 μg/kg dexmedetomidine infused intravenously over 5 min could significantly decrease the incidence and severity of sufentanil-induced cough. The principal mechanism was the ability of α2-adrenoreceptor agonists to counteract the effects of sufentanil-induced muscle stiffness. Liu et al. [5]found that applying 0.1 mg/kg dezocine intravenously before sufentanil injection could effectively suppress sufentanil-induced cough. By activating κ receptors, dezocine's suppression of cough may oppose opioid-activated μ receptors [21]. Nuanwan et al. [7] implied intravenous lidocaine 0.25 mg/kg for 2 min before fentanyl injection was the most effective dose to suppress fentanyl-induced cough(FIC) and could be applied in daily practice. It has been suggested that the way lidocaine works is by making the peripheral cough receptors in the trachea and hypopharynx anesthetic. The study by Saori et al. [22] showed that the swallowing action immediately before intravenous fentanyl may be a simple and clinically feasible method for effectively preventing FIC. They postulated that the process of swallowing could lower intrathoracic pressure and inhibit facial nerve injury. A previous study showed that low-dose ketamine could effectively reduce fentanyl-induced cough and delay the onset time of cough due to its bronchodilatory effects [23]. However, ketamine's use is currently restricted because of its hallucinogenic side effects and increased blood pressure, intracranial pressure, and intraocular pressure during general anesthesia, ketamine's use is currently restricted [24]. Compared to ketamine, esketamine exhibits less side effects and a greater sedative and analgesic efficacy as an N-methyl-D-aspartic acid (NMDA) receptor antagonist [10]. After intravenous injection of esketamine, the time to peak blood concentration of esketamine reached 1 min [25]. Therefore, the pretreatment drug was administered at 1 min before sufentanil injection in our study. The result of this study indicates that pretreatment with esketamine can effectively reduce the incidence and severity of sufentanil-induced cough. It has been documented that the larynx, lung, and airways contain NMDA receptors, and that airway constriction can result from these receptors being activated [9]. Esketamine may cause bronchiectasis by antagonistically binding to the NMDA receptor on the smooth muscle of the bronchi.

Our study has several limitations. We did not evaluate how esketamine dosages affected the frequency and intensity of coughs brought on by sufentanil. Furthermore, there was no esketamine pretreatment in this study to reduce coughing brought on by other opioid medications. More research is needed to determine how different esketamine doses impact the frequency and severity of sufentanil-induced cough during the induction of anesthesia. Furthermore, this research is a single center clinical trial. Consequently, in order to investigate the impact of esketamine on sufentanil-induced cough, extensive multicenter randomized controlled trials are required.

## Conclusion

In conclusion, this research indicates that pretreatment with 0.15 mg/kg esketamine significantly suppressed the frequency and intensity of sufentanil-induced cough. Therefore, pretreatment with a small dose of esketamine presents an effective way to prevent sufentanl-induced cough during the induction of anesthesia.

## Data Availability

The authors are pleased to share individual identified participant data. The data of this study including figures and tables will be available by contacting the corresponding author.

## Abbreviations

NBP: Noninvasive blood pressure
SpO2: Pulse oxygen saturation
ECG: Electrocardiogram
MAP: Mean arterial pressure
HR: Heart rate
